# Links Between Gambling and Academic Performance Among Undergraduate College Students: A Scoping Review

**DOI:** 10.1007/s10899-025-10440-9

**Published:** 2025-10-23

**Authors:** Hannah K. Allen, Tonya Knight, Becky Hall, Melissa Dennis

**Affiliations:** 1 William Magee Institute for Student Wellbeing, University of Mississippi, University, MS USA; 2 Department of Public Health, University of Mississippi, University, MS USA; 3 University of Mississippi Libraries, University of Mississippi, University, MS USA

**Keywords:** Gambling, Academic performance, College students, Scoping review

## Abstract

Gambling among young people is a growing public health concern, particularly as gambling becomes more accessible through changing policy and increased online gambling platforms. College students are a high-risk population for problem gambling, yet limited research has synthesized evidence on its academic implications. This scoping review examined the association between gambling behavior and academic performance among undergraduate college students in order to summarize findings, identify methodological patterns, and highlight gaps to inform future research. Eligible studies included peer-reviewed, empirical research that quantitatively assessed the relationship between gambling and academic performance (i.e., grades) among undergraduate college students worldwide. Thirteen studies met the inclusion criteria and were included in the review. The majority of studies found a negative association between gambling and academic performance in college, with both general gambling behavior and pathological gambling consistently linked to lower grade point average (GPA). There is a vital need for updated research in the context of a rapidly changing gambling landscape, as the majority of studies in this review were over ten years old. To address methodological limitations of existing research on the relationship between gambling and academic performance among college students, future research should prioritize longitudinal data collection, standardized measures of gambling behavior, and the use of rigorous statistical methods that account for potential covariates. Gambling may impair academic performance in college students, and additional research is needed to better understand this relationship and inform campus-based prevention, harm reduction, and treatment strategies to bolster student success.

## Introduction

Gambling is becoming increasingly prevalent and is an important public health priority. Over 70% of U.S. adults reported gambling at least once in the past year, and the number of people displaying risky gambling behavior increased from 7% in 2018 to 11% in 2021 (National Council on Problem Gambling, 2023). There are several key risk factors for problem gambling or gambling disorder, such as being male, young, single or married less than five years, living alone, having a low level of education, and having financial difficulties (Moreira et al., 2023). Gambling and gambling disorder have been linked to a multitude of consequences, including decreased quality of life, poor mental health, heavy substance use, financial issues, damage to social relationships, and adverse impact on work and education (Abbott, 2020; Moreira et al., 2023). By 2028, net gambling losses by consumers worldwide are expected to reach almost $700 billion (Wardle et al., 2024).

Increased attention is being paid to gambling in light of rapidly changing gambling policies and technologies worldwide, which are ultimately making gambling more accessible (Abbott, 2020). There has been a significant rise in online and mobile gambling, and the rapid and intensive nature of online gambling products increases prevalence, frequency, and potential harms (Marionneau et al., 2023; Tran et al., 2024). The commercial gambling industry has increased advertising and social media messaging to target consumers, utilizing consumer data to tailor marketing and increase engagement (Wardle et al., 2024). In the U.S. specifically, gambling is legal under federal U.S. law, with varying policies among states related to lotteries, casino gambling, online gambling, and sports betting. Almost all U.S. states allow some form of gambling, and about half of U.S. states have legalized online gambling and/or sports betting. The U.S. Supreme Court overturned the Professional and Amateur Sports Protection Act (PASPA) in 2018, which led 38 states and Washington D.C. to legalize retail sports betting and/or mobile-online sports betting. Approximately $6.6 billion was legally wagered on sports in the U.S. in 2018, and that number grew to $148 billion in 2024 (Bisson, 2025).

Young adults are a high-risk population for gambling problems. Only 24% of 18–24 year old gamblers show no signs of problematic play compared to 29% of 25–34 year olds, 35% of 35–44 year olds, 66% of 45–54 year olds, 85% of 55–64 year olds, and 91% of gamblers 65 years old and older (NCPG, 2023). College students in particular are a high-risk group given pervasive sports betting on college campuses and a lack of formal college and university policies related to gambling (Rychlak, 2023). Past-year gambling among U.S. college students is estimated to be 60%, although recent data is lacking (Caldeira et al., 2017). According to a 2023 study by the National Collegiate Athletic Association (NCAA, 2023), 60% of students pursuing a college degree engage in sports betting as compared to 54% of young adults not pursuing a degree. A meta-analysis by Nowak (2018) estimated that about 6% of college students are pathological gamblers and 10% engage in problem gambling behavior. Gambling disorder has been linked to mental health problems, substance use, and risky sexual behavior among college students (Grant et al., 2019).

Better understanding predictors of decreased academic performance among college students is vital, as long-term consequences of lower academic achievement and educational attainment include increased risk for functional limitations, disability, and overall poor physical and mental health (Zajacova & Lawrence, 2018). Several health factors have been identified as risk factors for decreased academic achievement among college students, including poor nutrition, sleep, and substance use habits (Lederer et al., 2024; Ong et al., 2021). There is an emerging body of literature on gambling as a potential behavior with links to academic performance among young people, with research showing longitudinal links between gambling participation and decreased academic performance among adolescents (Vitaro et al., 2018) and an association between problem gambling and lower grades among college and university students (Avenyo et al., 2024; Grant et al., 2019). Additionally, gambling and academic performance may be linked due to an overlap of risk factors and a correlation between gambling and other health characteristics, such as mental health and substance use problems, that have been shown to negatively impact academic success among college students (Caldeira et al., 2017; Grant et al., 2019).

There is a need to synthesize the current research literature on the relationship between gambling and academic performance in this high-risk population in order to inform future research and programming efforts on college campuses. Accordingly, this scoping review aims to identify existing peer-reviewed literature, summarize methodological approaches and study findings, and identify gaps to be addressed in future research studies.

## Methods

This review seeks to synthesize the current research available and identify gaps in the literature on the association between gambling and academic performance among undergraduate college students. This scoping review applied a PCC framework (population, construct, context) (Peters et al., 2020) and had the following primary research question: What is known from the literature about the association between gambling and academic performance among undergraduate college students?

The authors conducted this review in accordance with the Preferred Reporting Items for Systematic Reviews and Meta-Analyses (PRISMA) Extension for Scoping Reviews (Tricco et al., 2018) and deposited their review protocol in [redacted for review]. The scoping review was conducted in accordance with the Joanna Briggs Institute (JBI) methodology for scoping reviews (Peters et al., 2022).

### Inclusion and Exclusion Criteria

This review included studies with samples of undergraduate college students pursuing a bachelor’s degree or equivalent at a university or college in the U.S. and international settings. Studies focused on the association between gambling and academic performance among undergraduate college students. Gambling was defined broadly and included all gambling behavior assessed quantitatively, including gambling with and without experiencing gambling-related problems or gambling disorder. Studies had to use grade point average (GPA) or academic grades to objectively assess academic performance.

This scoping review only included empirical studies using human participants that were published in peer-reviewed journals with full text and written in English. Reviews, meta-analyses, theoretical studies, scale validation studies, and studies published in the grey literature were excluded.

### Search Strategy

The search was conducted on March 6, 2025, and included databases with broad subject coverage to identify articles from a variety of disciplines. Using the EBSCOhost platform, the authors searched Academic Search Premier, APA PsychInfo, Business Source Complete, CINAHL Plus with Full Text, Criminal Justice Abstracts, ERIC, and SPORTDiscus with Full Text. In addition, the database PubMed was searched separately. No date restrictions were set. See Table 1 for a list of search terms used.

**Table 1:** Database search terms

College students	Gambling	Academic performance
undergraduates	gambl*	academic achievement
college students	betting	academic success
university students	wagering	student success
	gaming	academic performance
		GPA
		grades
		grade point average

*Used to locate articles including words with this root (e.g., gamble, gambling, gambler)

Articles identified for review were exported to Rayann, a research collaboration platform, with duplicates removed. Remaining articles were independently screened by title and abstract for their relevance to the research question. At least two reviewers assessed each article to determine eligibility using the identified inclusion and exclusion criteria. After excluding articles that did not meet the inclusion criteria after title and abstract review, at least two reviewers assessed the full text of all remaining articles to determine eligibility for inclusion in this review. In addition, the reference lists of full-text articles screened were reviewed as an additional source of articles that may fit the inclusion criteria. If any articles were identified through reference lists, the full text was screened by at least two reviewers. During all phases of article screening, conflicts between reviewers were resolved through discussion with the entire research team. Study authors were contacted to clarify study methods or results if needed.

## Results

**Fig. 1:**
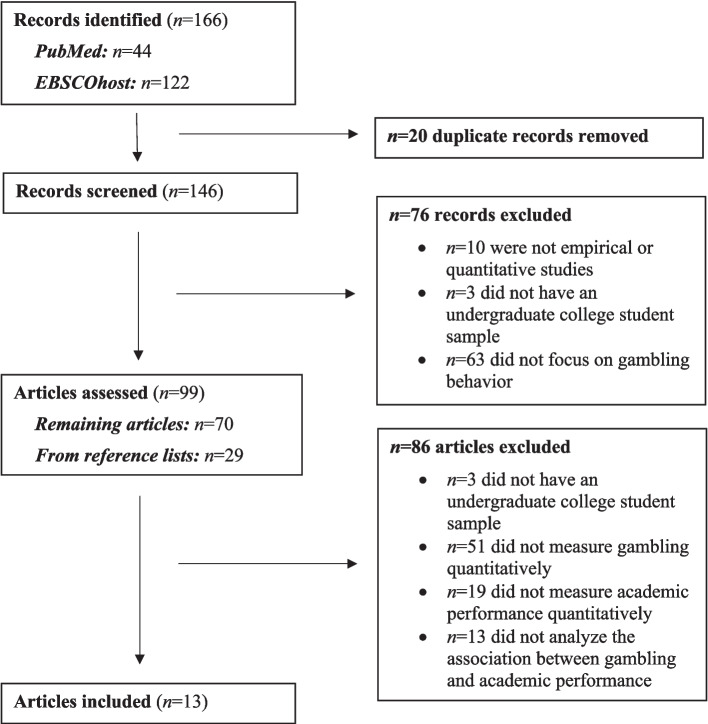
Scoping review methodology, based on PRISMA guidelines

Search outcomes are outlined in a PRISMA-ScR (PRISMA extension for Scoping Reviews) flow diagram (Fig. 1; Tricco et al., 2018). The initial database search yielded *n* = 146 unique records that were examined by title and abstract. After removing records that were not empirical or quantitative studies (*n* = 10), did not have an undergraduate college student sample (*n* = 3), and did not focus on gambling behavior (*n* = 63), the study team assessed the full text of the remaining *n* = 70 articles. During this process, an additional *n* = 29 articles were identified through reference lists. After removing *n* = 86 articles that did not meet inclusion criteria, *n* = 13 articles were selected for final inclusion in this scoping review.

Data extraction was conducted for the *n* = 13 articles selected for final inclusion in the scoping review for (a) publication details (e.g., study authors, year), (b) study site, (c) sample, (d) data collection methods, (e) measurement of gambling, (f) measurement of academic performance, and (g) findings related to the association between gambling and academic performance. This information is presented in Table 2.

**Table 2:** Included studies and results of data extraction (*n* = 13)

Authors (year)	Study site	Study sample	Measure of gambling	Measure of academic performance	Findings on the relationship between gambling and academic performance
Avenyo et al. (2024)	Ghana	*N* = 245 students from five universities	Online sports betting disorder, measured using an adapted version of the Game Addiction Scale (Lemmens et al., 2009)	Course grades	Negative relationship between online sports betting disorder and grades
George et al. (2016)	India	*N* = 5,580 students from 58 colleges	Lifetime gambling behavior and problem gambling, measured using the NODS-CLiP (Toce-Gerstein et al., 2009)	Failed a subject in exams	Problem gamblers had worse academic performance than both non-gamblers and non-problem gamblers
Grant et al. (2019)	U.S	*N* = 3,421 students from one university	Past-year problem gambling, measured using the Minnesota Impulsive Disorders Interview (Grant & Steinberg, 2005)	GPA	Problem gamblers had lower GPAs than students without a gambling problem
Harris et al. (2013)	Canada	*N* = 325 students from one university	Classified as lifetime non-gamblers, non-Internet gamblers, and Internet gamblers	Academic average	No significant findings
Kitole et al. (2025)	Tanzania	*N* = 258 students from three universities	Past-year sports betting	GPA	Negative relationship between sports betting and GPA
LaBrie et al. (2003)	U.S	*N* = 10,275 students from 119 universities	Classified as past-year non-gamblers or gamblers	GPA	Gamblers were more likely than non-gamblers to have a GPA of less than a B +
Lesieur et al. (1991)	U.S	*N* = 1,771 students from six universities	Pathological gambling, measured using the South Oaks Gambling Screen (Lesieur & Blume, 1987)	GPA	Negative relationship between pathological gambling and GPA
Petry & Weinstock (2007)	U.S	*N* = 1,356 students from three universities	Classified as never Internet gamblers, infrequent Internet gamblers, and frequent Internet gamblers	GPA	Internet gamblers had lower GPAs than non-Internet gamblers
Petry and Gonzalez-Ibanez (2015)	U.S	*N* = 117 students from three universities who were recent problem gamblers	Classified as not recent Internet gamblers or recent Internet gamblers	GPA	No significant findings
Sen and Pirincci (2024)	Turkey	*N* = 217 students from one university who gambled in their lifetime and provided data on GPA	Lifetime gambling behavior and problem gambling, measured using the South Oaks Gambling Screen (Lesieur & Blume, 1987)	GPA	Students with a GPA of less than 60 had higher scores on the South Oaks Gambling Screen than those with a GPA of 60 and above
Weinstock et al. (2008)	U.S	*N* = 159 students from one university who gambled in the past year	Pathological gambling, measured using the Diagnostic Interview for Gambling Severity (Winters et al., 1996)	GPA	Pathological gamblers had lower GPAs than non-pathological gamblers
Winters et al. (1998)	U.S	*N* = 1,361 students from two universities	Past-year pathological gambling, measured using the South Oaks Gambling Screen (Lesieur & Blume, 1987)	GPA	No significant findings
Yani-de-Soriano et al. (2012)	U.K	*N* = 209 students from multiple universities who gambled online in the past month	Classified as no problem online gamblers, at-risk online gamblers, and pathological online gamblers	Past-month academic problems, including low grades	Pathological online gamblers had the most academic problems, followed by at-risk online gamblers and no problem online gamblers

*U.S.* United States, *U.K.* United Kingdom, *GPA* Grade point average

### Methodological Features of Studies Meeting Inclusion Criteria

All included studies used a paper-and-pencil or online self-report questionnaire to collect data, with one study also reviewing academic transcripts (Avenyo et al., 2024) and one study also conducting diagnostic interviews (Weinstock et al., 2008). Nine of the studies collected data from students at multiple colleges and universities, with the remaining four studies collecting data from students at a single college or university. Study sample sizes varied widely and ranged from *n* = 117 students (Petry & Gonzalez-Ibanez, 2015) to *n* = 10,275 students (LaBrie et al., 2003). Four studies restricted their analytic samples by gambling behavior in some way, including students who had gambled in their lifetime (Sen & Pirincci, 2024), gambled in the past year (Weinstock et al., 2008), gambled online in the past month (Yani-de-Soriano et al., 2012), or who were recent problem gamblers (Petry & Gonzalez-Ibanez, 2015).

Gambling behavior, problem/pathological gambling, and gambling disorder were assessed and used in relevant analyses in the included studies. Gambling behavior was assessed at various time intervals (i.e., lifetime, past-year, past-month) and included a wide range of gambling activities (e.g., sports betting, casino games, lottery, cards). Several studies (*n* = 5) focused specifically on online gambling behavior (Avenyo et al., 2024; Harris et al., 2013; Petry & Weinstock, 2007; Petry & Gonzalez-Ibanez, 2015; Yani-de-Soriano et al., 2012), and two studies focused specifically on sports betting behavior (Avenyo et al., 2024; Kitole et al., 2025).

Problem/pathological gambling and gambling disorder were assessed using various scales, including an adaptation of the Game Addiction Scale (Lemmens et al., 2009), the NODS-CLiP (Toce-Gerstein et al., 2009), the Minnesota Impulsive Disorder Interview (MIDI; Grant & Steinberg, 2005), the DSM IV-TR-Based Questionnaire (Beaudoin & Cox, 1999), the South Oaks Gambling Screen (SOGS; Lesieur & Blume, 1987), the Canadian Problem Gambling Index (CPGI; Ferris & Wynne, 2001), and the Diagnostic Interview for Gambling Severity (DIGS; Winters et al., 1996).

The majority of the studies assessed academic performance using academic average, grade point average (GPA), or college course grades. The exceptions were George et al. (2016), who used failing a subject in exams as a measure of academic performance, and Yani-de-Soriano et al. (2012), who assessed academic performance using a sum score of presence of seven different academic problems in the past month, including low grades.

Statistical methods varied, with the majority of studies (*n* = 9) using bivariate analytic methods only to test group differences or to explore the association between gambling and academic performance without accounting for potential covariates. Only *n* = 4 studies included potential covariates when examining the association between gambling and academic performance (Avenyo et al., 2024; Kitole et al., 2025; LaBrie et al., 2003; Lesieur et al., 1991). Of note, LaBrie et al. (2003) and Lesieur et al. (1991) conducted initial bivariate tests of correlation before introducing GPA into multiple regression models predicting gambling behavior.

### The Association between Gambling and Academic Performance

When exploring gambling behavior, Kitole et al. (2025) found that sports betting had a significant, negative association with GPA, and LaBrie et al. (2003) found that having a GPA of less than a B + was more common among students who gambled in the past year as compared to those who had not. However, GPA was not a significant predictor of past-year gambling when introduced into a multiple regression model by LaBrie et al. (2003).

Three studies assessed the association between online gambling and academic performance. While Petry & Weinstock (2007) found that students who gambled online had significantly lower GPAs than students who did not gamble online, two other studies found no significant associations. Harris et al. (2013) found no significant difference in academic average when comparing non-gamblers, non-online gamblers, and online gamblers, and Petry and Gonzalez-Ibanez (2015) found no significant difference in GPA when comparing problem gamblers who gambled online with problem gamblers who did not gamble online.

The majority of studies (*n* = 8) assessed problem/pathological gambling or gambling disorder, and several of these studies compared academic performance among students grouped by gambling status. Four studies found that problem/pathological gamblers had significantly worse academic performance when compared to non-gamblers and/or non-problem gamblers (George et al., 2016; Grant et al., 2019; Weinstock et al., 2008; Yani-de-Soriano et al., 2012). Sen and Pirincci (2024) found that lifetime gamblers with worse GPAs had significantly higher scores on the SOGS, and Lesieur et al. (1991) found that pathological gambling had a significant, negative bivariate association with GPA. However, when GPA was introduced into a multiple regression model predicting SOGS scores in the Lesieur et al. (1991) study, it was no longer statistically significant. Only one study (Winters et al., 1998) found no significant association between academic performance and pathological gambling.

Avenyo et al. (2024) focused specifically on online sports betting among college students and found a significant, negative association between online sports betting disorder and academic performance. This study was also unique in that the researchers explored whether the association between online sports betting disorder and academic performance varied by gender, and they found no sex-specific differences. Similar findings related to gender differences were noted by LaBrie et al. (2003), who did not find sex-specific differences in the association between past-year gambling and GPA.

This review focused on studies that used objective measures of academic performance and excluded studies that only assessed other academic behaviors (e.g., missing class, time spent studying) or measures of the self-reported impact of gambling on academic behavior or performance. However, it should be noted that *n* = 5 of the included studies used additional academic measures in their analyses, yielding relevant findings to the research question.

Harris et al. (2013) asked students who had gambled online if their online gambling negatively affected their academic achievement and if they missed classes because they were engaging in online gambling. While most online gamblers in the study did not agree with these statements, endorsing these statements was significantly associated with problem gambling as measured by a composite of their scores on the DSM IV-TR-Based Questionnaire, the SOGS, and the CPGI. For students classified as problem gamblers, Petry and Gonzalez-Ibanez (2015) found that those who engaged in online gambling missed school due to gambling more often than those who did not engage in online gambling.

LaBrie et al. (2003) found that studying for less than three hours a day was associated with past-year gambling, as was considering academics to be less than very important (but only among male students). While Lesieur et al. (1991) reported that 4% of their college student sample cut classes to gamble and 2% had lost time from work or school due to gambling, no analyses beyond descriptive statistics were performed. Finally, Yani-de-Soriano et al. (2012) used a measure of academic performance that included seven items, including reduced study hours, missing classes, and failing to submit an assignment. A sum score for academic performance was computed, and results showed that pathological gamblers reported the most academic problems as compared to at-risk gamblers and no-problem gamblers.

## Discussion

This scoping review identified 13 published empirical studies that examined the association between gambling and academic performance among undergraduate college students. With a few exceptions, there was a consistent pattern of negative associations found between gambling and academic grades in this population. While this body of literature is still developing, there appear to be academic consequences to gambling during college that need to be addressed through continued research, programming, and policy.

Over half of the studies included in this review found that college students who engaged in problem or pathological gambling had significantly worse academic performance than their non-gambling or low-risk peers (Avenyo et al., 2024; George et al., 2016; Grant et al., 2019; Lesieur et al., 1991; Sen & Pirincci, 2024; Weinstock et al., 2008; Yani-de-Soriano et al., 2012). However, it also appears that engaging in any gambling, regardless of experiencing gambling-related problems or meeting criteria for gambling disorder, is negatively associated with academic performance (Kitole et al., 2025; LaBrie et al., 2003). Several mechanisms may explain these observed associations between gambling and decreased academic performance among college students. Students who engage in gambling may spend less time engaging in behaviors conducive to academic success, such as attending classes, studying, and completing assignments. Prior research has supported this theory, finding that gambling is associated with decreased study time (LaBrie et al., 2003), and problem gambling is associated with increased academic problems such as failing to submit assignments and missing classes (Yani-de-Soriano et al., 2012).

There may also be underlying risk factors, such as impulsivity and sensation-seeking, that are associated with both gambling (Celik et al., 2022; Grant et al., 2019) and poor academic performance among college students (Henning et al., 2022). Additionally, co-occurring mental health and substance use issues may exacerbate the association between gambling behavior and poor academic performance in this population (Caldeira et al., 2017; Grant et al., 2019). The results of a mediation analysis by Kurilla et al. (2022) found that while gambling problems were not directly linked to poor class attendance among college students, alcohol use problems mediated the relationship between these two variables. Gambling losses may also cause financial stress for students, which may lead to students taking on additional employment at the expense of their academic engagement.

The existing literature on gambling and academic performance also provides insight into specific forms of gambling, including online gambling. The availability of mobile apps and real-time betting enables continuous gambling engagement, which could interfere with academic performance among college students. However, findings from existing research are mixed. Avenyo et al. (2024) found that engaging in online sports betting at a disordered level was linked to poor academic performance, and Petry and Gonzalez-Ibanez (2015) found no significant difference in GPA between problem gamblers who gambled online with problem gamblers who did not gamble online. Taken together, these findings may suggest that problem or disordered gambling, whether the behavior is online or not, has negative implications for academic performance. While Petry & Weinstock (2007) found that online gamblers had lower GPAs than non-online gamblers, Harris et al. (2013) found no significant difference in academic performance between non-gamblers, non-online gamblers, and online gamblers. One potential explanation for these disparate findings is methodological differences between the two studies. Petry & Weinstock (2007) assessed GPA among students at three universities, and Harris et al. (2013) assessed academic average among students at only one university. Additionally, while both studies split the sample into three groups based on gambling behavior, Petry & Weinstock (2007) classified students as never Internet gamblers, infrequent Internet gamblers, and frequent Internet gamblers, while Harris et al. (2013) classified students into lifetime non-gamblers, non-Internet gamblers, and Internet gamblers. Future research should continue to explore online gambling as a unique correlate of academic achievement.

There were methodological limitations to the studies included in this review that provide important areas for future research. The included studies were cross-sectional, so no conclusions can be drawn regarding potential causal or bidirectional links between gambling and academic performance among college students. However, research conducted in adolescent populations has found longitudinal associations, with poor academic performance as both a predictor and consequence of gambling over time (Dowling et al., 2017; Vitaro et al., 2018). While inclusion criteria limited the measure of academic performance to GPA and academic grades, studies varied widely in their measurement of gambling. Definitions and measurement of gambling ranged from broad behavioral classifications to specific diagnostic scales, and standardized tools should be prioritized in future research to improve comparability across studies. Additionally, the majority of the studies were published more than ten years ago, indicating a need for more recent research given the ever-changing gambling landscape.

A noteworthy gap in the existing literature is the limited use of multivariate models to adjust for potential covariates when exploring the relationship between gambling and academic performance in college student samples. Bivariate associations between gambling and academic performance were no longer significant in multivariate models in two studies (LaBrie et al., 2003; Lesieur et al., 1991), suggesting that this relationship may be explained by covariates such as demographic characteristics, personality traits, health status, and health behavior. Only two studies included in this review examined differences in the association between gambling and academic performance by gender (Avenyo et al., 2024; LaBrie et al., 2003), and neither found sex-specific differences. Future research should employ more rigorous statistical methods to isolate the unique contribution of gambling to academic outcomes among college students as well as explore the potentially moderating effects of sociodemographic characteristics such as age, gender, and race.

This scoping review should be interpreted in light of its limitations. This review only included studies published in peer-reviewed journals and written in English, which may have excluded relevant findings published in other languages or in the grey literature. While this review followed a rigorous scoping review methodology, no statistical conclusions can be made about the association between gambling and academic performance among college students. Although multiple reviewers were involved in each step of screening and followed a clear set of inclusion and exclusion criteria, there is a chance for reviewer bias based on subjective decision-making. This scoping review provides an overview of the existing literature on this topic, and future research should include a meta-analysis of study results on the association between gambling and academic performance among college students.

While all included studies examined gambling and academic performance among college students, the majority of the studies used the terms “college students” or “university students” to describe their samples without specifying if the sample included only undergraduate students or both undergraduate and graduate students. One included study (Grant et al., 2019) specifies that the sample included college and graduate students, and their results on the association between gambling and academic performance were not presented separately for students at various levels of study. Inability to discern between the two student populations in the included literature could potentially limit the specificity of the findings. Graduate students are a unique campus population, and future research should explore the links between gambling and academic performance in this subset of students.

Additionally, this review did not include studies conducted among students enrolled at community colleges, despite prior research finding that community college students have a higher risk for problem gambling than students enrolled at four-year universities (Sherba & Gersper, 2017). This scoping review also excluded studies that only used qualitative methods or that only assessed academic behaviors besides grades (e.g., missing class, time spent studying). Not only are these important areas of future research, but there may be informative findings from existing studies using these methods that were not included in this review.

This scoping review highlights a small pool of existing research on the association between gambling and academic performance among college students, with the majority of studies finding a significant, negative association. These findings point to the potential academic harms of gambling during a critical developmental period and highlight the need for greater research and institutional attention to gambling as a public health and student wellbeing issue. Future research should prioritize longitudinal data collection, including ecological momentary assessment of daily gambling and academic behaviors, as well as consistent measurement strategies and inclusion of relevant covariates to better understand the mechanisms linking gambling to academic outcomes. Research should continue to explore various forms of gambling (e.g., sports betting, online gambling) and their links to academic performance in college, as well as subpopulations of students who may be at increased risk for academic consequences associated with their gambling.

Findings suggest a need to integrate gambling education, screening, and harm reduction strategies into student wellness initiatives, particularly in light of the rapid expansion of legalized gambling and targeted advertising aimed at youth. As gambling becomes increasingly prevalent among young people, understanding and mitigating its academic consequences will be essential to promote student success during college and health and well-being across the lifespan.
